# Right lung complete atelectasis: an endotracheal tube displacement complication

**DOI:** 10.1093/omcr/omab085

**Published:** 2021-09-13

**Authors:** Apostolos Dimos, Andrew Xanthopoulos, Filippos Triposkiadis

**Affiliations:** Department of Cardiology, University Hospital of Larissa, Larissa, Greece

## Abstract

A 78-year-old, overweight woman with a severe individual history of the cardiovascular system was admitted in the intensive care unit with acute pulmonary edema. Despite appropriate emergency treatment, the patient did not show any clinical improvement and emergency intubation was decided. Post-intubation physical examination revealed dullness to percussion, absent breath sounds and reduced chest excursion of the right hemithorax combined with a gradual drop in blood pressure and oxygen saturation. An emergency chest X-ray showed opacification of the entire right lung and an ipsilateral shift of the mediastinum. Improvement of the patient’s respiratory and hemodynamic status was observed immediately after the partial withdrawal of the tube. Tube displacement is a relative frequent complication and concerns mainly the right main bronchus due to anatomical procedures. However, the above case is a rare case of tube displacement in the left main bronchus, which led to total atelectasis of the rightlung.

A 78-year-old woman with a history of heart failure (LVEF = 50%), hypertension and Parkinson’s disease was admitted to ICU with acute pulmonary edema associated with new-onset slow-atrial fibrillation. A temporary pacemaker was implanted and intravenous diuretic treatment was started. The patient subsequently developed respiratory failure (Fig. 1a) and was emergently intubated. An 8-cm endotracheal tube was inserted and secured at 22 cm from central incisors. Five-point auscultation technique and post-intubation bag-mask ventilation confirmed the proper position of the endotracheal tube. Ten minutes later, unexplained hypoxia appeared (pH = 7.33; pO_2_ = 61 mmHg; pCO_2_ = 72 mmHg; HCO_3_^−^ = 38 mEq/L; S_a_O_2_ = 89%) despite the high oxygen delivery (FiO_2_ = 100%) accompanied by a gradual blood pressure drop (BP = 90/45 mmHg). The physical examination revealed dullness to percussion, absent breath sounds and reduced chest excursion of the right hemithorax. A suction test revealed no mucous or secretions. An emergency chest X-ray showed opacification of the entire right lung and an ipsilateral shift of the mediastinum (Fig. 1b). Patient’s respiratory (pH = 7.47; pO_2_ = 83 mmHg; pCO_2_ = 49 mmHg; HCO_3_^−^ = 35 mEq/L; S_a_O_2_ = 97%) and hemodynamic status improved immediately after the partial (4 cm) withdrawal of the tube. A second chest X-ray demonstrated expansion of the right lung (Fig. 1c).

**Figure 1:**
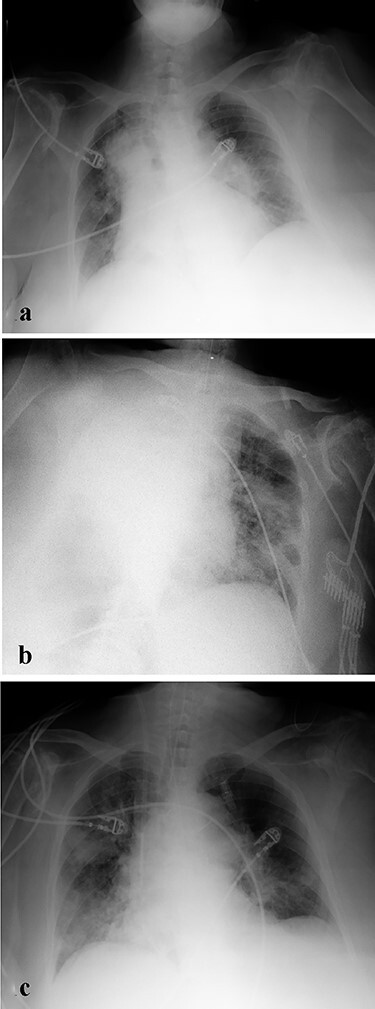
(**a**) X-ray depicting acute pulmonary edema in a critically ill patient shortly before intubation. (**b**) First post-intubation X-ray of the patient after displacement of tracheal tube in the left main bronchus. Atelectasis of the entire right lung. (**c**) Second post-intubation X-ray of the patient after withdrawing tracheal tube 4 cm. Expansion of the rightlung.

Tube displacement is a relative frequent complication (~13%) in critically ill patients undergoing tracheal intubation who manifest obstructive atelectasis (<1% of intubations lead to total lung collapse/atelectasis). Other causes include neoplasm, mucus plug or foreign body [1–3]. Risk factors of tube displacement include long duration of endotracheal intubation, absence of a muscle relaxant and intubation during cardiac arrest [4]. Symptoms range from mild cough to severe dyspnea and agitation, while clinical examination reveal decreased mobility in the affected lung area, dullness on percussion, and absent breath sounds [2]. Improper head and neck manipulations during and immediately after intubation may cause tube displacement, typically observed in the right main bronchus, due to anatomical reasons [5, 6]. Following standard intubation protocols and performing post-intubation radiography is mandatory to avoid unfavorable clinical scenarios such as complete lung atelectasis and hemodynamic instability.
